# Oral health-seeking behavior among different population groups in Enugu Nigeria

**DOI:** 10.1371/journal.pone.0246164

**Published:** 2021-02-01

**Authors:** Nkolika Uguru, Obinna Onwujekwe, Chibuzo Uguru, Udochukwu Ogu, Chinenye Okwuosa, Chinyere Okeke

**Affiliations:** 1 Faculty of Dentistry, Department of Preventive Dentistry, College of Medicine University of Nigeria Enugu, Enugu, Nigeria; 2 Health Policy Research Group, Department of Pharmaco-therapeutics, College of Medicine, University of Nigeria, Enugu, Nigeria; 3 Faculty of Health Sciences and Technology, Department of Health Administration and Management, University of Nigeria, Enugu, Nigeria; 4 Faculty of Dentistry, Department of Oral and Maxillo-facial Surgery, College of Medicine, University of Nigeria, Enugu, Nigeria; 5 Faculty of Medical Sciences College of Medicine, Department of Community Medicine, University of Nigeria, Enugu, Nigeria; University of Zurich, SWITZERLAND

## Abstract

**Introduction:**

This study investigates the oral health-seeking behaviour of households and its influence on demand for dental caries treatment services in Enugu state Nigeria.

**Methods:**

A quantitative descriptive cross-sectional study was used to explore the oral health seeking pattern of 378 urban and 348 rural household respondents in Enugu state Nigeria. The study explored dental caries treatment-seeking, oral health behavior of respondents using the three dynamics of the Andersen and Newman health utilization model; predisposing, enabling and need factors.

**Findings:**

Recommendations from community members (48.9%), severity of disease (22.1%), and cost of treatment (19.4%) all influenced where oral healthcare was first sought. Gender and type of occupation, influenced positive oral health-seeking behavior (p<0.05). The least poor socioeconomic status (SES) group, sought dental treatment in the private dental clinics, while the very poor and most poor SES groups used traditional healers, home treatment and patent medicine dealers more. Dental fillings and extractions were generally the most accessed treatment options for dental caries. The tendency for all the SES groups (especially the least poor), to choose tooth extraction more as a treatment option for dental caries was influenced by the oral health awareness level of respondents and the cost of dental fillings. (p<0.05).

**Conclusion:**

The findings suggest that interventions to create increased oral health awareness targeted at education on preventive strategies, appropriate time and place to seek oral health care and dental caries treatment, as well devising and implementing health financing options such as dental insurance would enable individuals to seek appropriate treatment for dental caries on time. In addition, it will reduce the proportion of people visiting unorthodox healthcare providers for their oral health problems or choosing cheaper but inappropriate treatment options.

## Introduction

Healthcare seeking behavior (HSB) is, any action or inaction, taken by individuals who believe that they have a health problem or believe that they are ill [1]. It comprises activities carried out to maintain good health, to prevent ill health, as well as any departure from a good state of health [2]. Different authors have used varied models to explain health-seeking behavior, however, the Health Belief Model, as well as Andersen's Health Behaviour Model have aptly described the series of steps people take towards accessing health care [3,4]. These models focus on an individual's belief about their health condition and the perceived consequence of the individual's action or inaction.

The factors that significantly affect health-seeking behavior during illness episodes and also, the utilization of the formal or informal oral healthcare facilities and self/home care remedies by individuals seeking oral health care, have been described in several studies [5–7]. These studies have shown that the decision to seek care through a particular process or from a particular provider is influenced by a variety of factors such as socioeconomic status (SES), sex, age, severity and type of illness, access to services and perceived quality of the service [6–8]. Besides, geographic location, family size, educational status, and cultural factors are influencers of oral health-seeking decisions in Nigeria [8–11]. The process of decision making for healthcare is complicated and might entail seeking the counsel of friends and family members, weighing potential risks and benefits, and forgoing other types of consumption that can be financed with resources used to purchase oral health care [11]. Thus, oral healthcare-seeking seems to be inextricably linked to the demand for oral healthcare services [12]. Thus the demand for oral healthcare services is often associated with an individual's choice about which service to access and when and where to access healthcare services [13].

Oral diseases as a key element of overall health has created huge public health concern mostly because of their significant impact on the quality of life of individuals, as well as the health system [14]. Dental caries and periodontal diseases are the most common oral diseases in the world and have been found to affect a large proportion of any population, making it a public health issue [15]. Dental caries is one of the most important contributors to the global oral health burden [16], and affects 60 to 90% of school children and almost 100% of adults worldwide [17]. It is estimated to affect 3.5 billion people globally and 2.3 billion people globally, suffer from caries of permanent teeth [18]. However, treating oral health conditions has been known to be expensive and despite this, not usually a part of universal health coverage [18]. The prevalence of oral diseases continues to increase in most of the low and middle-income countries with increasing urbanization and changes in living conditions [18].

In Nigeria, the prevalence of dental caries ranges from about 4 to 40% in adults [19,20], and the prevalence of untreated dental caries is 90% [21], affecting disadvantaged people [22], who are less likely to receive dental care or seek dental care in good oral healthcare facilities [17,23]. Studies in Enugu state Nigeria, have also recorded similar patterns with the prevalence of untreated dental caries cases at 97% [24]. The oral health utilization pattern for dental services in Enugu state show urban-rural disparity with 15.5% utilization in rural areas [24,25] and 65.4% in urban areas [26]. Another study conducted in Enugu state showed that uptake of regular scaling and polishing is low amongst the low socio-economic status groups and respondents living in rural areas [27]. This is as a result of poor oral health awareness, low financial power, political and health system challenges such as inadequate number and maldistribution of skilled human resources for oral health, inadequate dental facilities, and inadequate funding for oral health [19–22,25–27].

Studies investigating oral health-seeking behaviors have observed that inappropriate HSB has been linked to worse oral health outcomes, increased morbidity and mortality, and poorer oral health statistics [28,29]. These studies also show that not all community members, especially in rural areas or the poorer SES groups, receive information about oral health, or receive adequate oral health-promoting measures [28,29]. The decision to seek appropriate oral healthcare, therefore, lies on the respondent. Thus, to better understand the determinants of demand for dental caries treatment services, further insight into respondent's oral health-seeking behavior would be an added advantage.

This study thereby sought to identify the factors that affect an individual's decision to seek oral health care and to choose from among different providers. An understanding of oral health-seeking behavior will enable policymakers understand the drivers of demand for oral health services and inform the implementation of appropriate oral health strategies to improve access.

### Conceptual framework

The Andersen and Newman healthcare utilization model, a proposed framework for assessing healthcare utilization, was adapted to categorize and better explain the factors identified in this study that influenced oral health seeking behaviour [30]. This model posits that three dynamics determine the usage of health service i.e. predisposing factors (preexisting individual characteristics), enabling factors and need factors [30]. The predisposing factors include: educational level, marital status, gender, occupation, Health beliefs (attitudes, awareness of oral health care delivery systems); Enabling factors include: income, social or community network, SES); Need factors, include: severity of illness necessitating care, perceived health status, level of pain from the tooth).

## Materials and methods

### Study area

The study was conducted in Enugu state in the southeastern part of Nigeria. Enugu state is divided into 17 Local Government Areas (LGAs). Four urban and thirteen rural LGA's [31]. The state has a projected population of 4,411,100 with an urban population of about 1,032,297 and rural population of 2,235,540 [32]. The urban population is made up of mainly civil servants, traders, government workers, professionals, artisans, and students of the various educational institutions in the state while the rural population is made up of both artisans, subsistence farmers, traders as well as academics and government workers by virtue of the university campus located in the vicinity [31]. Two local government areas (one urban and one rural) were the study sites in the State. Enugu East, an urban LGA with a projected population of 374,100 and Nsukka, a rural LGA with a projected population of 417,700 [33]. This study was conducted at the household level to enable the collection of demand-side information from respondents to support analysis.

### Study design

A descriptive cross-sectional study design using a quantitative approach was employed for this study. The survey explored the demand side functions, such as, cost of dental caries treatment services, oral health-seeking patterns, episodes of dental caries, cost of care, attitudes, opinions, and oral health knowledge of study respondents. It was used to establish a relationship between selected variables and the demand for oral health services.

### Study population

The study population comprised of household residents in the selected study settings. All residents in the selected households who had dental caries within six months from the time of the study were included in the study. Six months was selected as the recall period to coincide with the stipulated six-monthly dental checkup visit. Households, where no member had suffered an episode of dental caries within six months from time of study and households outside the selected local government areas, were excluded from the study.

### Sampling

A prevalence of 56.3% obtained from the literature on dental healthcare utilization in an urban setting was used to estimate the sample size for the community survey. Where: $n=\frac{Z^{2}\mathrm{PQ}}{D2}$ n = the desired sample size when the population is greater than 10,000, Z = the standard normal deviate, usually set at 1.96 which corresponds to 95% confidence level, P = prevalence of utilization of dental services, and Q = 1-p. The same formula was also used for rural households. However, a prevalence of 34.8% found in a study conducted in Edo state on access and utilization of dental care in a rural area was used instead [34,35]. The sample size obtained from the formula for the urban and rural areas was 378 and 348 respectively.

A multi-stage sampling technique was used to select eligible households for the survey. The first stage involved the selection of local government areas (LGAs) in urban and rural areas respectively where the public dental facilities offering primary dental services were situated. This was purposively selected based on the location of the primary dental facilities.

At the second stage, a list of political wards for Nsukka and Enugu East LGA was obtained from the Department of Planning, Research and Statistics, Enugu State Ministry of Health. Each of the two LGAs comprised 20 political wards [36]. Three political wards from each of the two LGAs were selected randomly. Each of the political wards had 15 streets. Thirdly, each household in the streets and communities were visited, and households with an episode of dental caries within 6 months of our study were selected until our sample size was achieved. Households with members that had dental caries experience were identified and given a unique code and included in the study. Household heads in the selected households were interviewed to determine those that had ever experienced dental caries. In every selected household, the head of the household or representative was the respondent and the household was the unit of analysis.

### Data collection

The household questionnaire was developed through a review of the literature and informed by articles and studies conducted on access to oral healthcare services. Close-ended questions on awareness, health-seeking patterns, use of dental facilities, oral hygiene practices, socioeconomic status, cultural and social norms were asked. Information on respondents’ socio-demographic characteristics, previous dental experience, income level, cost of treatment services, insurance coverage, cultural issues, were also collected. Information on accessibility to oral health services, causes of inaccessibility and inequalities in access, were also collected during the interviews.

Household quantitative interviews that took into consideration respondents' socio-economic status data (household income, assets and expenditure), gender, ethnicity, occupation, employment status were conducted to also determine socio-economic status groups of respondents. Data was collected by 4 trained researchers and the questionnaires were administered to the respondents by the researchers.

### Ethical considerations

All respondents gave informed consent before the interviews were conducted. The respondents were given full information about the study, and were given an opportunity to ask questions before signing the consent form. Consequently, the interviewee’s consent to participate in the study was demonstrated by signing the Consent Form. In addition to the consent form, ethical clearance for the study was obtained from the ethical review board of the University of Nigeria Teaching Hospital, Enugu.

### Validity and reliability of instrument

The study instruments were validated using content validity, to determine whether a representative sample of the behavior domain to be measured was covered. It entailed the studying of research instruments to ensure adequate representativeness of actual observations, this was certified by an expert in the field. To test for the reliability of the study instrument, 20 questionnaires were distributed to 2 other groups of respondents with similar characteristic features to the main study respondents. Results obtained were seen to be similar.

### Data analysis

Data Analysis was conducted with SPSS version 23 and STATA 12 software. Frequency and percentages were computed as well as a test of association between dependent and independent variables. Chi-square and multiple logistic regression were used to determine the test for associations and correctly predict our outcome. All tests of significance were carried out at a p-value <0.05. Data were presented in tables and narratives as shown in the result section.

The dependent variables used in the study explored whether an individual had experienced any oral health problem six months prior to the study, whether they visited a dental clinic for dental caries treatment or any other oral health problem. This described where respondents first sought care for dental caries treatment. A set of independent variables namely (1) Socio-demographic variables (sex, age, marital status, education status) was used in the study. Education status was categorized into educated or not educated. Educated was further categorized according to the level of education achieved. (2). Sought treatment for a dental problem was described with a categorical answer: Yes or No. (3). Reason for seeking dental care was categorized as (a) pain, (b) have swollen gum, (c) difficulty in chewing, (e) mouth odour. (4). Types of treatment received by respondents were categorized as (a) dental filling, (b) extraction, (c) root canal treatment. (5). Respondents’ perception of ill health described as the severity of oral health problem which may influence oral health seeking behaviour was used. Furthermore, the SES index was used to categorize household respondents into SES quintiles: poorest, very poor, most poor, poor, and least poor. Principal components analysis (PCA) was used to generate the SES index [37]. The input to the PCA was information on ownership of key assets such as a motorcar, a motorcycle, a radio, a refrigerator, a television set, a gas cooker, iron, generator, and a bicycle together with the per capita cost of food. The SES index was disaggregated into quintiles with Q1 as the poorest and Q5 as the least poor.

## Results

Table 1 below, showed that 42.1% of the respondents were male, while 57.9% were female. 51.9% of the respondents belonged to the 21 to 40 years age group, while a majority of the respondents (57.4%) had completed senior secondary school. Most of the respondents (31.8%) were petty traders, and 6.7% were subsistence farmers.

**Table 1:** Socio-economic/demographic characteristics of respondents.

Variables	Rural n (%) N = 380	Urban n (%) N = 394	Total n (%) N = 774
**Sex**			
Male	198(25.6)	128(16.5)	326(42.1)
Female	182(23.5)	266(34.4)	448(57.9)
**Age (Years) of the household head or representative**			
Below 20	18(2.3)	15(1.9)	33(4.3)
20 to 40	152(19.6)	250(32.3)	402(51.9)
41 to 60	167(21.6)	120(15.5)	287(37.1)
Above 60	43(5.6)	9(1.2)	52(6.7)
**Marital status**			
Single	99(12.8)	127(16.4)	226(29.2)
Married	266(34.4)	238(30.7)	504(65.1)
Divorced/Widowed/Separated	15(1.9)	29(3.8)	44(5.8)
**Religion**			
Christianity	378(48.8)	392(50.6)	770(99.5)
Islam	2(0.3)	2(0.3)	4(0.5)
**Respondents’ highest level of education**			
Primary	13(1.7)	45(6.0)	58(7.7)
Junior secondary	9(1.2)	42(5.6)	51(6.8)
Senior secondary	183(24.4)	248(33.0)	431(57.4)
Tertiary	155(20.6)	56(7.5)	211(28.1)
**Main Occupation**			
Unemployed	67(5.0)	67(8.7)	134(17.3)
Subsistence farmer	39(5.0)	13(1.7)	52(6.7)
Petty trader*	58(7.5)	188(24.3)	246(31.8)
Government worker	84(10.9)	34(4.4)	118(15.2)
Private sector worker	30(3.9)	21(3.5)	57(7.4)
Self- employed professional**	90(11.6)	44(5.7)	134(17.3)
Artisan***	12(1.6)	21(2.7)	33(4.3)
**Mean no. in household (STD)**	5.32 (1.700)	3.98 (1.395)	4.64 (1.689)
**Socio-Economic Status groups**			
Q1 (Poorest)	76(20.1)	79 (20.1)	157(20.3)
Q2 (Very poor)	75(19.8)	79 (20.1)	153(19.8)
Q3 (Most poor)	76(20.1)	79 (20.1)	155(20.0)
Q4 (Poor)	76(20.1)	79 (20.1)	155(20.0)
Q5 (Least poor)	76(20.1)	78 (19.8)	154(19.9)

*petty trader–daily subsistence small scale trading **Self-employed professional–middle to large scale business owner **Artisan–skilled manual worker.

Table 2 showed that the major dental problems experienced by respondents in the study such as pain (95.7%), hole in the tooth (91.3%) were similar across both geographic locations (p<0.05). Respondents’ in rural areas sought treatment in private dental clinics and used home remedies more than urban residents who visited the public dental facilities and patent medicine dealers (PMD) more (p<0.05). Majority of respondents across geographic location cleaned their teeth once a day.

**Table 2:** Oral health seeking pattern and perception of need.

Variables	Total n (%) N = 774	Rural n (%) N = 380	Urban N = 394	X^2^(P-value)
**Household member with a dental problem**				
Yes	774(100.0)	380(49.1)	394(50.9)	0.966 (0.326)
**Sought treatment for a dental problem**				
Yes	769(99.4)	377(48.7)	392(50.6)	0.239(0.681)
No	5(0.6)	3(0.4)	2(0.2)	
*Reasons for seeking dental care				
Pain	741(95.7)	354(45.7)	387(50.0)	12.160 (0.001)
Swollen gum	317(41.1)	209(27.1)	108(14.0)	61.961 (0.001)
Difficulty in chewing	684(88.4)	343(44.3)	341(44.1)	2.598 (0.117)
Mouth odour	88(11.4)	50(6.5)	38(4.9)	2.369 (0.141)
Bleeding gums	205(26.5)	138(17.8)	67(8.7)	37.047 (0.001)
Shaking teeth	258(33.3)	225(29.1)	33(4.3)	224.94(0.001)
Hole in tooth	707(91.3)	334(43.2)	373(48.2)	11.230 (0.001)
Broken tooth	179(23.1)	44(5.7)	135(5.7)	55.992 (0.001)
Discoloured teeth	54(7.0)	3(0.4)	51(6.6)	44.033 (0.001)
Routine dental checkup	4 (0.6)	4(0.6)	0(0.0)	
Others	-	-	-	-
**How often do you clean your teeth**				
Not everyday	37(4.8)	36(9.5)	1 (3)	
Once daily	597 (77.1)	206(54.2)	391(99.2)	2.224 (0.001)
Twice daily	140 (18.1)	138(36.3)	2 (5)	
**Where the respondent sought treatment**				
Private dental clinic	286(37.0)	277(35.8)	9(1.2)	41.400 (0.001)
Public dental facility	306(39.6)	33(4.3)	273(35.3)	29.720 (0.001)
Pharmacy	2(0.3)	2(0.3)	0(0.0)	2.079(0.149)
Traditional healer	7(0.9)	3(0.4)	4(0.5)	0.110 (0.740)
Home treatment	56(7.2)	56(7.2)	0(0.0)	62.592 (0.001)
Hospital	2(0.3)	7 (0.9)	0(0.0)	7.324 (0.007)
Patent medicine dealer	110(14.3)	2(0.3)	108(14.0)	11.472(0.001)
**Factors that influenced where respondents sought treatment**				
Cheaper cost	150 (19.4)	48(6.2)	102(13.2)	21.758 (0.001)
Severity of dental problem	171(22.1)	153 (19.8)	18(2.3)	158.923(0.001)
Recommendation from friend	378(48.9)	140(18.1)	180(23.3)	6.238 (0.013)
Qualification of provider	36(4.7)	24 (3.1)	12(1.6)	4.664 (0.031)
Closeness to house	15(1.9)	6(0.8)	9(1.2)	0.506 (0.477)
Previous dental experience	21(2.7)	6(0.8)	15(1.9)	3.638 (0.056)
Staff attitude	3(0.4)	3(0.4)	0(0.0)	3.123 (0.077)

*Multiple responses.

Table 3 below shows a statistically significant relationship between, gender, type of occupation, dental symptoms and seeking dental treatment at a dental clinic (p<0.05). The male respondents were less likely to visit the dental clinic than the female respondents. In addition, self-employed professionals, government workers and petty traders were more likely to go to the dental clinic for dental treatment compared to the unemployed. Respondents that brush twice daily are 0.8 times more likely to visit a dental facility than those that don’t brush daily, but CI includes 1 so the variable was not significant to the model. Respondents without pain, were 0.2 times less likely to visit a dental clinic than those with pain and respondents in poorest SES group were less likely to go to the dental clinic than those in least poor SES group.

**Table 3:** Multiple logistic regression analysis between socio-economic and demographic factors associated with seeking care in a dental facility.

Independent Variables	Regression coefficient (b)	Adjusted OR	Confidence Interval		p-value
			Lower limit	Upper limit	
**Geographic location**					
Urban (ref)	0	1			
Rural	-0.298	0.742	0.412	1.337	0.321
**Gender**					
Female	0	1			
Male	-0.442	0.643	0.431	0.959	0.03
**Age group**					
Below 20	-0.558	0.573	0.148	2.210	0.418
21–40	0.088	1.092	0.408	2.925	0.86
41–60	-0.087	0.917	0.358	2.350	0.856
Above 60 (ref)	0	1			
**Marital status**					
Single (ref)	0	1			
Married	0.122	1.13	0.686	1.861	0.632
**Level of education**					
Primary	-0.155	-0.857	0.342	2.145	0.741
Junior secondary	0.165	1.218	0.513	2.89	0.655
Senior secondary	0.353	1.423	0.828	2.444	0.202
Tertiary (ref)	0	1			
**Main occupation**					
Unemployed (ref)	0	1			
Self-employed	1.519	4.569	1.477	14.133	0.001
Government worker	1.641	5.159	1.471	18.1	0.01
Petty trader	1.203	3.331	1.108	10.014	0.032
Subsistence farmer	0.885	2.423	0.748	7.852	0.14
Artisan	1.014	2.755	0.87	8.726	0.085
**Awareness**					
Do not brush daily(ref)	0	1			
Brush twice a day	0.221	0.802	0.257	2.498	0.033
**SES quintiles**					
Q1 (Poorest)	-0.692	0.501	0.277	0.906	0.022
Q2 (Very Poor)	-0.524	0.592	0.336	1.044	0.07
Q3 (Most Poor)	-0.273	0.761	0.432	1.339	0.343
Q4 (Poor)	-0.47	0.625	0.35	1.115	0.112
Q5 (least poor) (ref)	0	1			
**Dental symptoms**					
Yes symptoms (ref)	0	1			
Pain	-1.686	0.185	0.041	0.833	0.028
Shaking (mobile) teeth	1.029	2.799	1.582	4.954	0.001
Hole in teeth	0.845	2.329	1.143	4.745	0.02

*Dependent Variable: Did you visit a dental clinic for your dental problem?

Table 4 below shows that majority of the respondents (61.9%) had tooth extractions as a type of treatment for dental caries and this is influenced by the geographic location of respondents (p<0.05). 23% of respondents had dental fillings done with a greater percentage being in rural areas (p<0.05).

**Table 4:** Dental caries treatment received and determinants of the type of treatment received by respondents at a dental facility.

Variables	Total	Rural n(%)	Urban n(%)	Chi-square (p-value)
***Type of treatment received***				
Dental filling	178(23.0)	116(15.0)	62(8.0)	23.895(0.001)
Extraction	479(61.9)	257(33.2)	222(28.7)	10.447(0.001)
Root canal treatment	2(0.3)	2(0.3)	0(0.0)	2.079(0.241)
Crown	14(1.8)	0 (0.0)	14(1.8)	14.783(0.001)
Denture	17(2.0)	16(2.1)	1(0.1)	14.098(0.001)
***Determinants of the type of treatment received at the dental facility***				
Personal experience	118(15.2)	63(8.1)	55(7.1)	1.027 (0.311)
Experience of friend/family	285(36.8)	147(19.0)	138(17.8)	91.384(0.291)
High Cost of procedure	4(0.5)	3 (0.4)	1 (0.1)	1.080 (0.299)
Cheaper alternative	176(22.7)	65(8.4)	111(14.3)	13.486 (0.001)
Personal preference	169(21.8)	100(12.9)	69 (8.9)	8.783 (0.003)
Knowledge of procedures	18(2.3)	15 (1.9)	3(0.4)	8.644 (0.003)
Health insurance coverage	4(0.5)	3(0.4)	1(0.1)	1.080 (0.299)

Table 5 shows that the least poor SES group tend to seek dental treatment in the private facilities more than the poorest SES (26.7%). The poorest and very poor SES groups used home treatment and traditional healers more for dental treatment than the least poor SES.

**Table 5:** Level of inequities in utilization of dental caries treatment services across socioeconomic status groups.

Variables	Q1	Q2 n (%)	Q3 n (%)	Q4 n (%)	Q5 n (%)	Conc. Index	Chi-square	P-value
***Utilization pattern (Type of treatment Done)***								
Dental filling	30 (16.9)	37 (20.8)	38 (21.3)	41(23.0)	32(18.0)	0.02	3.137	0.535
Extraction	92 (19.2)	88 (18.4)	104(21.7)	85(17.7)	110(23.0)	0.03	12.951	0.012
***Where the respondent sought treatment***								
Private dental facility	34 (11.9)	41 (14.4)	72 (25.3)	62(21.8)	76(26.7)	0.15	39.823	0.001
Public dental facility	84 (27.5)	73 (23.9)	53 (17.3)	48(15.7)	48(15.7)	-0.12	27.954	0.001
Traditional healer	2 (28.6)	2 (28.6)	1 (14.3)	1 (14.3)	1 (14.3)	-0.18	0.855	0.931
Home treatment	2 (28.6)	2 (28.6)	1 (14.3)	1 (14.3)	1 (14.3)	-0.18	0.855	0.931
Hospital	16(28.6)	21(37.5)	9 (16.1)	4 (7.1)	6 (10.7)	-0.28	19.601	0.001
Patent medicine dealer	34 (30.9)	30 (27.3)	16 (14.5)	13(11.8)	17(15.5)	-0.19	18.213	0.001
***Factors influencing where treatment is sought***								
Cheaper price	45 (30.2)	33 (22.1)	22 (14.8)	27(18.1)	22(14.8)	-0.14	14.681	0.005
Severe dental problem	51 (29.8)	43 (25.1)	25 (14.6)	28(16.4)	24(14.0)	-0.16	21.354	0.001
Recommendation from friends/family	129(34.1)	90 (23.8)	58 (15.3)	54(14.3)	47(12.4)	-0.21	1.162	0.001
Provider qualification	16 (44.4)	2 (5.6)	7 (19.4)	4 (11.1)	7 (19.4)	-0.17	16.21	0.003
Closeness to house	4 (26.7)	2 (13.3)	5 (33.3)	2 (13.3)	2 (13.3)	-0.11	2.639	0.62
Previous dental experience	9 (42.9)	2 (9.5)	5 (23.8)	3 (14.3)	2 (9.5)	-0.26	8.213	0.084
Staff attitude	2(66.7)	0(0)	1 (33.3)	0(0)	0(0)	-0.54	5.427	0.263

Dental filling and extraction were the most used treatment options for dental caries treatment. The least poor (Q5) SES groups had tooth extractions done more than the other SES groups (p<0.05), while the poor (Q4) SES group had more dental fillings done. The concentration index shows very little income-related inequality. Cheaper treatment cost, the severity of dental problem and family recommendations, influenced where the poorest SES group sought treatment the most.

## Discussion and conclusion

Our study showed that all respondents had one form of oral health problem or the other and a majority had dental caries necessitating treatment. This lends credence to observations from other studies where dental caries was found to carry a high global oral health burden [14,15,17]. Using the Andersen and Newman’s healthcare utilization model, we discussed our findings based on the three dynamics of healthcare utilization mainly predisposing, enabling and need factors. The major predisposing factors to oral health seeking observed in this study include: gender, occupation, oral health awareness: We observed a gender influence on oral health-seeking, where the females visited the dental facility for treatment more than the males. The regression analysis buttresses this fact showing that females are more likely to visit the dental facility more than males. Women are the natural caregivers and as such are likely to seek healthcare treatment for every member of the family. Their frequent attendance at health facilities, often lead to a higher oral health awareness level than the males, which support their oral health-seeking behaviour for dental caries treatment [26]. Self-employed professionals and government workers made more visits to the dental clinic when compared with the unemployed. This can be associated with their financial capability and possibly increased awareness about oral health diseases. The fact that most government workers or professionals are likely to be under health insurance, will increase the likelihood of them visiting a dental facility.

Our logistic regression analysis shows that the geographic location and socio-economic status group though not statistically significant, have a negative regression coefficient implying that any increase in socioeconomic status will possibly result in an increase in the demand for treatment in a dental facility. In addition, any movement towards the urban area will result in an increase in the demand for dental caries treatment in a dental facility. This is similar to findings that showed higher dental service usage for adolescents who moved to better SES [38,39].

The enabling factors that influenced where dental caries treatment was sought include cost of services, qualification of provider, income, social or community network. This study showed that respondents' choice of provider was influenced by recommendations from friend/family members, and cost of services which always stands out as the determining factor for health-seeking [37,39]. Thus, oral health-seeking and decision about what type of treatment to receive could be said to depend on the counsel and recommendations of family and friends and the cost of services. Tooth extraction seemed to be the most preferred treatment sought by study respondents from both public and private clinics.

Results show that the poorest SES, sought dental treatment in the public facilities more than the least poor SES. This is because the cost of dental caries treatment services was cheaper in public facilities. Cost and availability of service concerning SES also influenced where treatment was sought. This conforms with the practice in other countries like China, where the private facilities were patronized by adults from higher-income households [41]. The poorest SES were also found to use home treatment and seek traditional healers more than their least poor counterparts. From this finding we can deduce that the poorer SES will usually seek the treatment they can afford first in non-dental facilities and if the disease increases in severity or cannot be appropriately managed by cheaper unorthodox means, they will then seek more expensive orthodox treatment. It could also be a pointer to their level of oral health awareness. As stated in the findings, the major mode of payment for health services was out-of-pocket and none of the respondents from the rural area had health insurance. This is not surprising, however, as a small number of respondents in the urban center have health insurance. Households bear a greater portion of these costs and other indirect costs with little or no financial risk protection. To some households, this may be catastrophic [40,41]. This calls for a need to provide some sort of financial risk protection mechanisms such as dental health insurance as this will have a positive impact on oral health-seeking behaviour of households. The oral health care system needs to be strengthened to function more effectively and decrease overall consumer economic burden if universal health coverage is to be achieved.

Dental filling and extraction are the most utilized treatment options for dental caries treatment. The rural respondents seemed to do more dental fillings and tooth extractions than the urban dwellers, thus suggesting an association between type of treatment with geographic location. However, it is important to note that there is a constant disintegration of social and economic boundaries between urban and rural areas [42], and in the context of this study, a university campus is located on the boundary of this rural area and as such, both low income and middle-income group workers who work in the institution and or make a living from the presence of the institution tend to settle more in the rural study area surrounding the institution and commute to work, possibly, to reduce household expenditures. Thus, it is difficult to associate utilization of a dental facility purely by geographic location.

Amongst the SES groups, the least poor seemed to opt more for extraction than the other SES groups. However, this can be explained by the fact that most respondents visited the dental facility when they were in severe pain, necessitating extraction or more advanced dental treatment such as root canal. The treatment choice of the poorest SES groups was influenced more by the cheaper treatment option. The financial impact of dental caries treatment was felt by all SES groups since most payment was out of pocket. However, we can deduce that the poorest SES group felt the impact more because of their low-income bracket. This is a clear indication that the poor will suffer more economic, financial, and intangible burden of oral health disease than the rich.

Need factors, include: severity of illness necessitating care, perceived health status, level of pain from a tooth. The severity of dental pain, dental swellings and other dental symptoms were observed to increase visit to a dental facility. Our findings show that majority of the respondents only went to the dental clinic when there was severe pain. Usually, an individuals’ perception of health will determine if the individual seeks care. Our study shows that the severity of dental pain is a major influencer of oral health-seeking behaviour.

In conclusion, females, those who are self-employed & government workers were more likely to use a dental facility for dental caries treatment. Cost of services, qualification of provider, social or community network such as recommendation from friends and family are enablers of oral health-seeking and treatment of dental caries in a dental facility. Severity of dental pain or dental condition affecting functionality such as tooth mobility or aesthetics such as tooth discoloration, influence the need to seek oral health services in a dental facility. It's important to note that the introduction and use of dental health insurance may reduce cost constraints for oral health-seeking in dental facilities.

The clinical implication for primary health care is that most dental complications could be prevented if oral health promotion activities to increase awareness were increased. This will equally reduce the high demand for dental caries treatment which is expensive. It will also promote self-care and increase the utilization of primary health care for the production of better health at lower costs.

### Study limitations and strength

Due to the fact that at the time of the study, there were no available documents or records on households that have had oral health problems and sought oral care, the researchers had to go from house to house mapping out homes that fit the study category. This meant that we had to cover a vast number of households before achieving the desired sample size.

As a strength, the findings and discourse of this study will go a very long way to charting a course into understanding oral health-seeking behaviour in Nigeria.

## Supporting information

S1 Data (XLSX)

S1 File Houshold (questionaire) instrument. (DOCX)

S2 File Variable response legend Oral health seeking. (DOCX)
