# Supplementary material for: Oral health-seeking behavior among different population groups in Enugu Nigeria
Source: PLoS One. 2021 Feb 1;16(2):e0246164. doi: 10.1371/journal.pone.0246164 (PMC7850484; doi:10.1371/journal.pone.0246164)
Supplement: S1 File — (DOCX) [file pone.0246164.s002.docx]

**DEMAND AND SUPPLY ANALYSIS OF INEQUITIES IN ACCESS TO DENTAL CARIES TREATMENT SERVICES IN ENUGU STATE NIGERIA**

***HOUSEHOLD QUESTIONNAIRE***

Good day Madam/Sir My name is ----------------------I am a trained data collector from College of Medicine, University of Nigeria, Enugu Campus. The purpose of this interview is to find out how you access and use oral health services for dental caries treatment. Dental caries or tooth decay as its commonly called is the most common oral disease worldwide and it causes a lot of pain and disability. This disease is highly preventable and if treated early the associated pain and disability can be averted. One of the common reasons for untreated dental caries is poor access to dental care which includes prevention and treatment of dental caries. We would therefore like to find out your knowledge about dental diseases especially dental caries, where you seek for treatment and if there are any challenges to accessing oral care. This will help us identify the level of demand for dental caries treatment services and inequities if any that exist in accessing care

Be assured that your identity will be kept confidential during the research process. Your participation is voluntary and you do not have to answer any questions you do not want to. However, the information that you will provide will help in achieving the aim of the study. If you have any questions you may ask them now or later.

***(Enumerator: Prior to beginning the interview, read the consent form on the last page to the respondent. Obtain a signature and Sign that they have read the form and consented)***

***Instruction: Please fill the next section at the beginning of the interview.***

***Interview Information***

This part should be filled by the enumerator before the interview

**1. Date:** |__|__||__|__||__|__|__|__|

**Name of Data collector: ______________________________________________**

**2. Geographic location: Urban [ ] Rural [ ] (**Rural =1 ; Urban =2)

**3. LGA: ---------------------------**

**4. Ward --------------------------- 5. Ward code: |___|___|**

**6. Date of data entry** |__|__||__|__||__|__|__|__| Time interview started -----------------

| **Section 1: Demographics** | | | | | | | | | | |
| --- | --- | --- | --- | --- | --- | --- | --- | --- | --- | --- |
| 7. | | Household study ID: | | | | | | [ ] | | |
| **Household head or Primary caregiver (representative) information** | | | | | | | | | | |
| 8 | | Sex | | | | 1. Male  2. Female [ ] | | | | |
| 9 | | Age (as at last birthday) | | | | [ ] | | | | |
| 10 | | Marital status | | | | [ ] Single=0; Married = 1; Divorced =2;  Widowed =3; Seperated = 4 | | | | |
| 11 | | Religion | | | | [ ] Christianity=0, Islam=1, traditional religion=2,  Other = 3 | | | | |
| 12 | | Educational status (Did you go to school | | | | [ ] Yes =1 ; No =0 | | | | |
| 13 | | What was your highest completed educational level?  Primary = 1  Junior Secondary =2  Senior Secondary = 3  Tertiary = 4 | | | | [ ] | | | | |
| 14 | | What is your MAIN Occupation  14a.Unemployed  14b. Subsistence Farmer  14c. Petty trading  14d. Government Worker  14e. Employed in private sector  14f. Artisan  14g.Self-employed professional  14h. Others [ ] Please, specify: | | | | Yes =1 ; No =0  [ ]  _____________________________________ | | | | |
| 15 | | Number of people in the household including yourself | | | | [ ] | | | | |
| 16. | | Number of Adults above 18 yrs | | | | [ ] | | | | |
| 17 | | Number of children below 18 yrs | | | | [ ] | | | | |
| 18 | | Add Q16 and 17 | | | | [ ] | | | | |
|  | | **Section 2: Oral health awareness** | | | | | | | | |
| 19 | | Have you ever received information on how to prevent dental disease | | | | Yes = 1; No =0 [ ] | | | | |
| 20 | | If yes to Q19 how did you first receive information on how to prevent dental disease?  Parents  Family members  Friends  Media  Doctor  Oral health outreach programs  Others | | | | Yes =1 ; No =0  [ ]  [ ]  [ ]  [ ]  [ ]  [ ]  [ ] Specify ----------------------------- | | | | |
| 21 | | How many times a day do you clean your teeth | | | | [ ]  once = 1  Twice =2  Three times = 3  None =4 | | | | |
| 22 | | What do you MOSTLY clean your teeth with?  Water = 1  Salt = 2  Chewing stick = 3  Tooth brush = 4  Toothbrush and paste =5  Charcoal =6  Herbal stick = 7  Chewing stick and paste =8  Other = 9 | | | | [ ]  If other, specify ------------------------ | | | | |
| 23. | | Have you or any member of your family ever gone for routine dental checkup | | | | [ ] Yes =1 ; No =0 | | | | |
| 24 | | How many times do you go in a year  Once a year [ ]  Twice a year [ ]  Three times a year [ ]  Other [ ] specify ------------------------- | | | | [ ] Yes =1 ; No =0 | | | | |
|  | | **Section 3: Healthseeking pattern and perception of need** | | | | | | | | |
| 25 | | In the last six months have you or any member of your household had any dental problems? | | | | [ ] Yes =1 ; No =0 | | | | |
| 26.  26a.  26b.  26c.  26d.  26e.  26f.  26g  26h  26i | | Can you tell me what the symptoms were  Pain  Swollen gum  Difficulty in chewing  Mouth odor  Bleeding gums  Shaking teeth  Hole in tooth  Broken tooth  Discolored teeth  Others | | | | Yes =1 ; No =0  [ ]  [ ]  [ ]  [ ]  [ ]  [ ]  [ ]  [ ]  [ ]  Specify ----------------------------------- | | | | |
| 27 | | Did you or any member of your family seek treatment for this dental problem? | | | | Yes =1 ; No =0 [ ] | | | | |
| 28a | | Do you know where to seek for dental treatment? | | | | [ ] Yes =1 ; No =0 | | | | |
| 28b | | Have you visited any place for dental treatment? | | | | [ ] Yes =1 ; No =0 | | | | |
| 28c | | If no to Q28b, why? | | | |  | | | | |
| 29a | | Did you visit a dental clinic for your dental problem | | | | [ ] Yes =1 ; No =0 | | | | |
| 29b | | Where did you first seek care | | | | 29b(i). Dental clinic [ ]  29b (ii). Traditional healer [ ]  29b (iii). Home treatment [ ]  29b (iv). Hospital [ ]  29b (vi). Pharmacy [ ]  29b (vii). Patent medicine dealer [ ]  29b (viii). Other [ ] specify ………………… | | | | |
| 30. | | If yes to Q 29a Which dental facility did you go to for treatment  ***(Interviewer should elicit reasons for choice of facility: please document in field note)*** | | | | 30a. Public dental facility [ ]  30b. Private dental facility [ ] | | | | |
| 31 | | If answer to any other option in Q29 is yes, Interviewer should find out details of where respondent accessed care and why? | | | | (please document in field note) | | | | |
| 32  32a.  32b.  32c.  32d.  32e  32f  32g | | If answer to Q 29a is **No.**  Why would you not go to a dental clinic  I don’t know where to find one  Fear to consult a dentist  I don’t need dental treatment  I prefer home treatment  They are too expensive  Other | | | | [ ]  [ ]  [ ]  [ ]  [ ]  [ ] Specify -------------------------------------- | | | | |
| 33 | | How often do you and your family members use the dental clinic? ( 1= Yes; 0 = No) | | | | 31a. Regular checkup [ ]  31b. Only when I have toothache [ ]  31c. To clean my teeth [ ]  31d. Only when I have a hole in my teeth [ ]  31c. others [ ]  specify ----------------------------- | | | | |
| **These questions are specific for dental caries treatment services:** | | | | | | | | | | |
|  | | | | | | | | | | |
| 34 | In the last 6 months did you or any member of your family seek treatment for dental caries (hole in teeth)  (*Normal dental recall period is 6 months. As dental caries is a slowly progressive chronic disease 6 months recall is adequate*) | | | | | [ ] 1= Yes; 0=No | | | | |
| 35 | How many family members received treatment for dental caries in the last 6 months | | | | | [ ] | | | | |
| 36 | Where did you go to receive this treatment | | | | | 36a. private dental clinic [ ]  36b. Public dental facility [ ]  36c. traditional healer [ ]  36d. home treatment [ ]  36e. hospital [ ]  36f. pharmacy [ ]  36g. Patent medicine dealer [ ]  36h. other [ ] specify …………………………… | | | | |
| 37 | If treatment was received at a public dental facility which one of the facilities did you use | | | | | 1. Tertiary facility [ ] 2. Secondary facility [ ] 3. Primary facility [ ] 4. Other [ ] specify ------------------------ | | | | |
| 38 | What influenced your choice of where you went for treatment for dental caries?  Cheaper price of services  Severity of the problem  If recommended by someone I trust  Qualification of provider  Closeness to my house  Previous dental experience  Staff attitude | | | | | 1=yes, 0=no  [ ]  [ ]  [ ]  [ ]  [ ]  [ ]  [ ] | | | | |
|  | **House hold head should fill out column for HH member 1 and any details from other HH member should be contained in column 2 and 3 (if more than 3 HH members have dental caries collect information on back of sheet)** | | | | | | | | | |
|  |  | | | HH member 1  (Age = ) | | | HH member 2  (Age = ) | | | HH member 3  (Age = ) |
|  | Please write Yes=1 and No=0 | | | | | | | | | |
| 39  39a  39b  39c  39d  39e  39f  39g | What type of treatment did you or any member of your family receive at the dental clinic for dental caries? In the last 6 months | | | Dental Filling [ ]  Extraction [ ]  Root canal [ ]  Crown [ ]  Denture (removable) [ ]  Others [ ]  specify ----------------- | | | Dental Filling [ ]  Extraction [ ]  Root canal [ ]  Crown [ ]  Denture (removable) [ ]  Others [ ]  specify ----------------- | | | Dental Filling [ ]  Extraction [ ]  Root canal [ ]  Crown [ ]  Denture (removable) [ ]  Others  specify ------------------- |
| 40  40a.  40b.  40c.  40d.  40e.  40f.  40g.  40h.  40i.  40j.  40k | How much did the visit to the dental clinic cost.  (Please indicate cost of every item respondent paid for and add total. | | | Registration/card [ ]  Consultation [ ]  Drugs [ ] X-ray [ ]  Dental Filling [ ]  Extraction [ ]  Root canal [ ]  Crown [ ]  Bridge (fixed) [ ]  Denture (removable)[ ]  Others [ ]  Total [ ] | | | Registration/card [ ]  Consultation [ ]  Drugs [ ] X-ray [ ]  Dental Filling [ ]  Extraction [ ]  Root canal [ ]  Crown [ ]  Bridge (fixed) [ ]  Denture(removable)[ ] Others [ ]  Total [ ] | | | Registration/card [ ]  Consultation [ ]  Drugs [ ] X-ray [ ]  Dental Filling [ ]  Extraction [ ]  Root canal [ ]  Crown [ ]  Bridge (fixed) [ ]  Denture(removable)[ ] Others [ ]  Total [ ] |
| 41 | How much did you pay to transport yourself to and from the facility | | | [ ] | | | [ ] | | | [ ] |
| 41b | How many hours did you spend in the clinic? | | |  | | |  | | |  |
| 42 | **Total cost:** (cost of treatment + transport cost) | | |  | | |  | | |  |
| 43 | Did you request for the specific treatment type? | | | [ ] Yes =1 No = 0 | | |  | | |  |
| 44 | Did you request for an alternate type of treatment  Yes =1 No = 0 | | | [ ] | | |  | | |  |
| 45  45a.  45b | If answer to 43 is Yes; please give reasons  Yes =1  No = 0 | | | Recommended treatment too expensive [ ]  Others [ ]  Specify------------ | | | Recommended treatment too expensive [ ]  Others [ ]  Specify------------ | | | Recommended treatment too expensive [ ]  Others [ ]  Specify------------ |
| 46. | What influenced the type of treatment you or any member of your family received at the dental facility? | | | | | | | | | |
| 46a.  46b.  46c.  46d.  46e.  46d. | Yes=1  No=0 | | Personal experience [ ]  Experience of friend/family [ ]  High Cost of procedure [ ]  Cheaper alternative [ ]  Personal preference [ ]  Knowledge of procedures [ ]  Health insurance coverage [ ]  Others [ ]  Specify ------------------------ | | Personal experience [ ]  Experience of friend/family[ ]  High Cost of procedure [ ]  Cheaper alternative [ ]  Personal preference [ ]  Knowledge of procedures [ ]  Health insurance coverage [ ]  Others [ ]  Specify ------------------------ | | | | Personal experience [ ]  Experience of friend/family [ ]  High Cost of procedure [ ]  Cheaper alternative [ ]  Personal preference [ ]  Knowledge of procedures [ ]  Health insurance coverage[ ]  Others [ ]  Specify ------------------------ | |

| **Payment coping** | | |
| --- | --- | --- |
| 47 | To what extent has the illness affected the family financially? Yes=1, No =0 | [ ]47a. no impact  [ ]47b. little impact  [ ]47c. minor impact  [ ]47d. serious impact  [ ]47e. very serious impact |
| 48 | Where did the money come from to pay for these expenses?  ***(Multiple responses allowed)***  Yes=1, No =0 | - [ ]48a. Health insurance - [ ]48b. Employer - [ ]48c. Cutting down on other expenses - [ ]48d. Out of pocket - [ ]48e. Borrowing - [ ]48f. Selling assets - [ ]48g. Asking for donations from - friends and relatives   [ ]48h. Others, specify |
|  |  |  |

| **HOUSEHOLDS’ Socioeconomic status** |
| --- |
| 49. How much did your household spend to purchase food from the market in the past one week on the various items listed?   \| Item \| Quantity \| Amount \| Who purchased \| Buy food (Total amount in a week) \| \| --- \| --- \| --- \| --- \| --- \| \| 49a.Gari  49b.Beans  49cCassava  49d.Cooking oil  49e.Yam  49f.Rice  49g.Corn  49h.Fish  49i.Meat  49j.Vegetables  49k.Others (specify) \|  \|  \|  \|  \| \| 49l.Grand Total \|  \|   50. If the food items that your household produced and also consumed in the past one week were bought from the market, how much will they cost?   \| Item \| Quantity \| Amount \| Produced (Total amount) \| \| --- \| --- \| --- \| --- \| \| 50a.Gari  50b.Beans  50c.Cassava  50d. Cooking oil  50e.Yam  50f.Rice  50g.Corn  50h.Fish  50i.Meat  50j.Vegetables  50k.Others (specify) \|  \|  \|  \| \| 50l.Grand Total \|  \|   51: Total food value (***Enumerator add 49l +50l***) [ ]   \| **Now we would like to ask you about your household assets** \|  \| \| --- \| --- \| \| 52. From the assets that I am going to read out to you, could please tell me the functional ones that your household has? Please answer yes or no. 1 = yes 0 = No \|  \| \| 52a. Radio \| Radio[ ] \| \| 52b. Bicycle \| Bicycle[ ] \| \| 52c. Manual grinding machine \| Gmachine[ ] \| \| 52d. Motor cycle/tricycle \| Mcycle[ ] \| \| 52e. Motor car \| Mcar[ ] \| \| 52f. Cattle/Poultry \| Cattle/Pou[ ] \| \| 52g.Sewing machine \| Sewma[ ] \| \| 52h.Refrigerator/Freezer \| Ref./fre [ ] \| \| 52i.Electric/Gas Stove \| Ele./gsto. [ ] \| \| 52j.Electric Fan \| Ele.fan [ ] \| \| 52k.Electric Iron \| Ele.iron[ ] \| \| 52l.Television \| Tv [ ] \| |

| **Household Amenities** | | |
| --- | --- | --- |
| 53 | What kind of toilet facility does the household use?  Yes=1  No=0 | 53a. None/bush [ ]  53b. Flush toilet [ ]  53c. Pan/bucket [ ]  53d. Covered pit latrine [ ]  53e. Uncovered pit latrine [ ]  53. Other, [ ] specify ................... |
| 54 | How does your household dispose of refuse?  Yes=1  No=0 | 54a. Collected by refuse agency [ ]  54b. Burned by household [ ]  54c. Public provided dump [ ]  54d. Dumped elsewhere [ ]  54e. Buried by household [ ]  54f. Other, [ ] Specify ..................... |
| 55 | What is your MAIN means of disposing liquid waste?  Yes=1  No=0 | 55a. Through the sewerage system [ ]  55b Thrown onto the street/outside [ ]  55c. Thrown into the gutter [ ]  55d. Thrown into the compound [ ]  55e Other, [ ] Specify ..................... |
| 56 | What is the MAIN source of drinking water for this household?  Yes=1  No=0 | 56a. Inside taps in dwelling [ ]  56b. Public outdoor tap [ ]  56c. Borehole [ ]  56d. Protected/Covered well [ ]  56e. Uncovered well  56f. Purchased treated water [ ]  56g. River/pond/lake [ ]  56h. Other [ ] (specify)………………… |
| 57 | What is the occupancy status of the household?  Yes=1  No=0 | 57a. Owner [ ]  57b. Rent [ ]  57c. Use without paying rent [ ] |
| 58 | How many sleeping rooms does your household have? | _______ |
| 59 | What is the MAIN material of the roof of the house?  Yes = 1  No = 0 | 59a. Mud [ ]  59b. Thatch [ ]  59c. Wood [ ]  59d. Metal sheets [ ]  59e. Cement/concrete [ ]  59f. Roofing tiles [ ]  59g. Asbestos [ ]  59h. Mud plastered with cement [ ]  59i Mud covered with mat [ ]  59j.Other [ ] (specify)………………. |
| 60 | What is the MAIN material of the walls of the house?  Yes = 1  No = 0 | 59a. Mud/mud bricks [ ]  59b. Stone [ ]  59c. Burnt bricks [ ]  59d. Cement [ ]  59e. Wood/bamboo [ ]  59f. Iron sheets [ ]  59g. Cardboard [ ]  59h. Other [ ] (Specify)………………….. |
| 61 | What is the MAIN fuel used for cooking?  Yes = 1  No = 0 | 61a. Firewood [ ]  61b. Charcoal [ ]  61c. Kerosene/oil [ ]  61d. Electricity [ ]  61e. Crop residue/saw dust [ ]  60f. Animal waste [ ]  60g. Gas [ ]  60h. Other [ ] specify ……………… |
| 62 | What is the MAIN source of energy for lighting?  Yes = 1  No = 0 | 61a. Kerosene/oil/Shea butter [ ]  61b. Gas [ ]  61c. Electricity [ ]  61d. Generator [ ]  61e. Battery [ ]  61f. Candle [ ]  61g. Other [ ] specify…………………… |

**Thank you** Time interview ends ---------------------------------------
