# Supplementary material for: Oral health-seeking behavior among different population groups in Enugu Nigeria
Source: PLoS One. 2021 Feb 1;16(2):e0246164. doi: 10.1371/journal.pone.0246164 (PMC7850484; doi:10.1371/journal.pone.0246164)
Supplement: S2 File — (DOCX) [file pone.0246164.s003.docx]

Oral health seeking behaviour among different population groups.

Description of response to variables in Excel data file

1. **Location**

1 = rural

2 = urban

1. **Gender**

1 = male

2 = female

1. **Marital status**

0 = single

1 = married

2 = separated/ widowed/divorced

1. **Religion**

0 = Christianity

1 = Islam

2 = traditional religion

1. **Education**

0 = no

1 = yes

1. **Highest level of education**

1 = primary

2 = junior secondary

3 = senior

4 = tertiary

1. **Main occupation**

1 = unemployment

2 = subsistence farmer

3 = petty trader

4 = government worker

5 = private sector

6 = self-employed professional

7 = artisan

1. **How did you first hear?**

1= parents

2 = family member

3 = friends

4 = media

5 = doctor

6 = oral health programs

1. **How many times a day do you clean your teeth?**

1 = once

2 = Twice

3 = Not every day

4= Thrice

1. **What do you mostly clean your teeth with?**

1=Water

2=Salt

3= Chewing stick

4= Tooth brush

5=Toothbrush and paste

6=Charcoal

7= Herbal stick

8= Chewing stick and paste

9 = Dental powder

1. **Have you or any member of your family ever gone for routine dental checkup**
2. = no
3. = yes
4. **How many times do you go for routine dental checkup in a year?**

1= Once a year

2= Twice a year

3= Three times a year

4 = Don’t know

1. **In** **the last 6 months have you or** **any member of your household** **had any dental problems?**

0 = no

1= yes

1. **Can you tell me what the symptoms were? (Response for all symptoms below is 0 = no; 1=yes)**

[i] Pain

[ii] Swollen gum

[iii] Difficulty in chewing

[iv] Mouth odor

[v] Bleeding gums

[vi] Shaking teeth

[vii] Hole in tooth

[viii] Broken tooth

[ix] Discolored teeth

**Response for all questions and response options below is (0 = no and 1 = yes)**

1. Did you or any member of your family seek treatment for this dental problem?
2. Do you know where to seek for dental treatment?
3. Have you visited any place for dental treatment?
4. Did you visit a dental clinic for your dental problem
5. **Where did you first seek care**

1 = Dental clinic

2 = Traditional healer

3 = Home treatment

4 = Hospital

5 = Pharmacy

6 = Patent medicine dealer

1. **Which dental facility did you go to for treatment?**

1 = Public dental facility

2 = Private dental facility

1. **Why would you go to a dental clinic?**

1 = I don’t know where to find one

2= Fear to consult a dentist

3 = I don’t need dental treatment

4 = I prefer home treatment

5 = They are too expensive

1. **How often do you and your family members use the dental clinic?**

1 = Regular checkup

2 = Only when I have toothache

3 = To clean my teeth

4 = Only when I have a hole in my teeth

1. **In the last 6 months did you or any member of your family seek treatment for dental caries (hole in teeth)** (0=no; 1= yes)
2. **Where did you go to receive this treatment**

1 = Private dental clinic

2 = Public dental facility

3 = Traditional healer

4 = Home treatment

5 = Hospital

6 = Pharmacy

7 = Patent medicine dealer

1. **If treatment was received at a public dental facility which one of the facilities did you use?**

1 = Tertiary facility

2 = Secondary facility

3 = Primary facility

1. **what influenced your choice of where you went for treatment for dental carries**

1 = Cheaper price of services

2 = Severity of the problem

3 = If recommended by someone I trust

4 = Qualification of provider

5 = Closeness to my house

6 = Previous dental experience

7 = Staff attitude

**Response for all questions and response options below is (0 = no and 1 = yes)**

1. **What type of treatment did you or any member of your family receive at the dental clinic for dental caries? In the last 6 months**

[i] Dental Filling

[ii] Extraction

[iii] Root canal

[iv] Crown

[v] Denture (removable)

1. Did you request for specific type of treatment?
2. Did you request alternate type of treatment
3. **What influenced type of treatment received at dental facility**

[i] Personal experience

[ii] Experience of friend/family

[iii] High Cost of procedure

[iv] Cheaper alternative

[v] Personal preference

[vi] Knowledge of procedures

[vii] Health insurance coverage

1. **To what extent has the illness affected the family financially?**

[i] no impact

[ii] little impact

[iii] minor impact

[iv] serious impact

[v] very serious impact

**(31) Response to household assets and amenities is (0=no and 1=yes)**
